# Should We Re-think Regulations and Standards for Lighting at Workplaces? A Practice Review on Existing Lighting Recommendations

**DOI:** 10.3389/fpsyt.2021.652161

**Published:** 2021-05-13

**Authors:** Oliver Stefani, Christian Cajochen

**Affiliations:** ^1^Centre for Chronobiology, Psychiatric Hospital of the University of Basel, Basel, Switzerland; ^2^Transfaculty Research Platform Molecular and Cognitive Neurosciences (MCN), University of Basel, Basel, Switzerland

**Keywords:** lighting, workplace, standards, circadian rhythms, non-image forming effects of light

## Abstract

Nowadays lighting projects often include temporal variations of the light, both spectrally and in terms of intensity to consider non-visual effects of light on people. However, as of today there are no specific regulations. Compliance with common lighting standards that address visual aspects of light, often means that only little non-visually effective light reaches the eye. In this practice review we confront existing regulations and standards on visual lighting aspects with new recommendations on non-visual aspects and highlight conflicts among them. We conclude with lighting recommendations that address both aspects.

## Introduction

The advent of electric lighting made it possible to decouple working hours by means of shift work from times when daylight was available. Concomitant to alterations in working hours, sleep-wake times are very often irregular in shift workers thereby impacting on the endogenous circadian timing system with negative health consequences (1). Light as the principal synchronizer (i.e., Zeitgeber) of human circadian rhythms, is “seen” during the biological night while at work, which might lead to circadian rhythms disturbance such as shift work sleep disorder (SWD). SWD is a circadian rhythm sleep disorder characterized by insomnia and excessive sleepiness affecting people whose work hours typically occur during the habitual sleep period (1). In particular light with high proportions of short wavelengths in the blue spectral range in the evening and at night suppress the secretion of the night hormone melatonin, a marker of circadian rhythmicity in humans. Additionally to negative light effects during the night, low illuminances during the day can destabilize circadian rhythms (2).

Continuously disturbing the entrainment of endogenous circadian rhythms with external diurnal Zeitgeber rhythms with can weaken the regenerative ability of the organism (3, 4). Night work is associated with negative consequences for somatic and mental health (5) and persistent desynchronization of endogenous rhythms limits cognitive performance (6). Thus, with the increase in flexible working times, innovative lighting concepts that take particular account of non-visual lighting effects become particularly important.

With the increased time spent in buildings, the length of time during which people are exposed to high amounts of daylight decreases. Although, lighting standards for workplaces ensure that we can see well, specifications for artificial lighting correspond to twilight conditions outdoors (7). 500 lx of artificial light indoors corresponds to ~0.5% of the light on a cloudless day. Measurements at workplaces have shown that workers are usually exposed to illuminance of only 100 lx for more than 50% of the day (8), which fall far below the recent recommendation of daylight illuminance exposure (9).

## Visual Effects of Light – Regulations, Standards, and Energy Aspects

Where life safety is threatened (e.g., in emergency situations like building fires) interior lighting projects must comply with requirements imposed by regulations regarding minimum levels of illuminance. Different regions of the world approach regulations, recommended practices, and standards differently. For the design and operation of workplaces, the German “Technical Rules for Workplaces” (ASR) for example, reflect the state of the art for occupational medicine, occupational hygiene, as well as other reliable ergonomic findings for setting up and operating workplaces. According to the German Workplace Ordinance, workplaces must receive as much daylight as possible and be equipped with artificial lighting that is appropriate for the safety and health protection of employees. The Technical Rules for Workplaces ASR A3.4 (10) “Lighting” specify the requirements of the Workplaces Ordinance for setting up and operating the lighting in workplaces as well as the requirements for glare protection when exposed to sunlight. In Germany for example, an artificial lighting system for workplaces must meet requirements concerning illuminance, limitation of glare, color rendering, flickering, or pulsation as well as shadows. DGUV Informative Publication 215-210 “Natural and Artificial Lighting of Workplaces” of the German Social Accident Insurance (DGUV) offers also assistance to employers in implementing the ASR A3.4 “Lighting.”

Standards differ from regulations. In general, lighting professionals are expected to appraise each design situation and develop criteria for illuminance, color rendering quality, uniformity, correlated color temperature (CCT) etc., that are appropriate for a project, and although, there is no obligation to comply to standards, they provide valuable guidance. Workplace lighting in particular should consider these standards. One example is the German DIN EN 12464-1:2011-08 (11). This standard specifies the planning principles for lighting systems, but does not specify the requirements for the safety and health protection of employees at work. This standard gives illuminance recommendations for the task area, the immediate surroundings, background area, and for walls and ceilings. In a new draft (prEN 12464-1:2019), higher illuminances are recommended in order to allow for adjustments. In the new draft, the horizontal illuminance of 500 lx for workplaces e.g., is now specified as a minimum requirement. A higher value (1000 lx) is specified, which is to be used e.g., in rooms with elderly persons with lower eyesight. The demands on lighting quality are determined by the visual tasks that the human eye has to master. The classic quality features of lighting can be divided into three basic quality features, which are weighted differently depending on the use of the room and the desired appearance: Visualization, visual comfort, and visual ambience. The following applies:

- Visual performance is influenced by the illuminance and the limitation of direct and reflected glare.
- Good color rendering and a harmonious brightness distribution ensure visual comfort.
- Visual ambience is determined by CCT, light direction, and modeling (i.e., the distribution of light and shadows).

Good lighting systems are also characterized by energy efficiency. However, according to DIN EN 12464-1 (11), the quality of light should not be reduced for lower energy consumption.

The purpose of the Swiss SIA 2024 (12) is to standardize assumptions about room usage, in particular about personal occupancy, and equipment usage. These assumptions should be applied in calculations and verifications according to the standards of energy and building services engineering if there is no more accurate information available. The requirements are regarded as standard values for the design of plants or factories in an early planning phase. Finally, typical values are given for power and energy requirements in the areas of appliances, lighting, ventilation, etc.,

Further, important features are flicker-free lighting and the possibility of changing brightness and CCT. Luminaires that are too bright in the field of view can cause glare. Therefore, light sources must be shielded in a suitable way. Glare is a complex topic that cannot be discussed in rigorous detail in this work. Other work, such as the DIN EN 12464-1 should be referred to.

When working with PC screens, care should be taken that the luminance ratio between the working field and its immediate surroundings is no greater than 3:1. The luminance ratio between the work surface and the more distant surfaces should not exceed 10:1. Due to higher luminances and improved anti-reflective coatings, modern PC screens can tolerate much higher environmental luminance levels than their predecessors. DIN EN 12464-1 describes the permissible limit values for avoiding reflected glare. For screens with a luminance of L ≤ 200 cd/m^2^ luminances of up to 1500 cd/m^2^ are permissible for luminaires. For monitors with a monitor luminance L > 200 cd/m^2^ (typical for offices with good to very good daylight supply and correspondingly adapted flat screens) luminance values of up to 3000 cd/m^2^ are permissible. The ambient contrast ratio (A-CR) is a key metric to achieve a high image quality of displays when considering bright ambient lighting (13). While OLED (organic light-emitting device) based displays exhibit several attractive features, such as self-emission, high brightness, and a high contrast ratio, when operated under bright ambient light, most of the incident light is reflected and decreases the A-CR which makes the application under high illuminances difficult. Today there are several methods to eliminate reflected ambient light in OLEDs (e.g., by a circular polarizer or a destructive interference layer). A-CR is generally defined as

$$\begin{array}{l} ACR=\frac{L_{\text{on}}+L_{\text{ambient}}\cdot R_{\text{L}}}{L_{\text{off}}+L_{\text{ambient}}\cdot R_{\text{L}}} \end{array}$$

where *L*_*on*_ (*L*_*off*_) represents the on-state (off-state) luminance value of an LCD or OLED, and *L*_*ambient*_ is ambient luminance (14). *R*_*L*_ is the luminous reflectance of the display panel.

For a balanced luminance in the room all surfaces must be taken into account. Surface luminances can be determined by the reflectance of the surfaces and the illuminance on the surfaces. According to DIN EN 12464-1 recommended reflectances are:

- ceiling: 0.7 to 0.9
- walls: 0.5 to 0.8
- floor: 0.2 to 0.4

Regarding good color rendering it is commonly recommended to have a CRI of Ra > 80. We have recently found evidence for positive effects of a high CRI (Ra 97 vs. 80) on visual comfort, daytime wakefulness, well-being, and nighttime sleep (15). A newer and better system for evaluating a light source's color rendering property is IES TM-30-15. The fidelity index and the gamut index of IES TM-30-15 of the LED causing the before mentioned positive effects were Rf.97 and Rg.101, respectively, and for the poorer performing LED it was Rf.81 and Rg.94.

Without shadows objects are only two-dimensional images. Only the correct distribution of light and shadow guarantees that faces and gestures, surfaces, and structures can be easily recognized (11). A pleasant lighting climate is created when people, architecture, and room furniture are illuminated in such a way that shapes and surface structures are clearly visible. Distances can be easily estimated and orientation in the room is made easier. Good visual communication requires that faces are easily and quickly recognized. In areas where good visual communication is important, for example in offices and meeting areas, DIN EN 12464-1 recommends a higher average cylindrical illuminance of 150 lx. The cylindrical illuminance is the average of all vertical light on an imaginary cylinder. DIN EN 12464-1 cites “modeling” as an important quality feature for the perception of people and objects. Modeling is the relationship between cylindrical and horizontal illuminance and should be between 0.30 and 0.60.

The international WELL Building Institute (16) aims for advancing health and well-being in buildings. It also provides recommendations for lighting to support visual acuity and is mainly based on the American National Standards Institute (ANSI) and Illuminating Engineering Society (IES) RP-1-20 (17) standard and on the standard of the Ontario Ministry of Labour, Computer Ergonomics: Workstation Layout and Lighting (18).

At workstations or desks requirements are met when

1. The ambient lighting system is able to maintain an average light intensity of 215 lx or more, measured on the horizontal work plane. The lights may be dimmed in the presence of daylight, but they should be able to independently achieve these levels.
2. The ambient lighting system is zoned in independently controlled banks no larger than 46.5 m^2^ or 20% of open floor area of the room (whichever is larger).
3. If average ambient light is below 300 lx, task lights providing 300 to 500 lx at the work surface are available upon request.

The American National Standards Institute and Illuminating Engineering Society of North America. RP-1-20 provides recommended luminance ratios for offices:

1. Luminance ratios should not exceed 3:1 between a paper task and an adjacent visual display terminal.
2. For ceiling luminance ratios, 10:1 is the maximum acceptable ratio.
3. Luminance ratios should not exceed 10:1 between a task and a remote surface.

In the latest version of the WELL v2 pilot (Q1 2021) it is recommended that indoor spaces should comply with one of the lighting reference guidelines (IES Lighting Handbook 10th Edition, EN 12464-1: 2011, ISO 8995-1:2002(E) (CIE S 008/E:2001), or GB50034-2013).

## Non-Visual Effects of Light

It is only known since 2002 that the human eye has a third photoreceptor for processing ambient light in addition to the two classical photoreceptor types, the rods, and cones (19, 20). Effects of visible radiation, which are mainly controlled by this newly discovered photoreceptor, but make a minor contribution to classical visual information processing, are also called non-visual light effects. Berson and colleagues reported that those ganglion cells containing the photopigment melanopsin project almost exclusively to the nucleus suprachiasmaticus (SCN), the central pacemaker driving circadian rhythms. They are also able to transmit light signals into the cortex without the help of the classical photoreceptors. Thus, these ganglion cells have been termed “intrinsically photosensitive retinal ganglion cells (ipRGC)” with melanopsin maximally sensitive to visible short-wave radiation (21).

Provencio et al. (22) reported that a coarsely resolved network of photosensitive ganglion cells extends over the retina of mice, which has the task of detecting brightness. Later, it was found that these melanopsin-containing ganglion cells are distributed not only in the fovea but also over the entire retina with a density of 3–5 cells/mm^2^ and have their maximum concentration of 20–25 cells/mm^2^ in the area surrounding the fovea (20, 23). IpRGCs are not evenly distributed across the retina but have a higher density in the lower half so that light that falls into the eye from above and impinges the lower half of the retina suppresses the nocturnal release of melatonin more than light from below (24, 25).

While blue-enriched light can contribute to increased alertness, especially in the evening and at night, nocturnal exposure to light reduces melatonin secretion. Melatonin secretion is especially reduced by light at night if people are exposed to only a low dose of light during the day (26). Today, people often live their lives in isolation from their natural environment with a high risk to develop circadian disorders (27). Severe circadian disturbances are found in people who are forced to chronically change their lifestyle by adapting their sleep-wake schedules to the imposed work schedules (e.g., rotating shift workers) (1). A milder form of circadian disorder occurs in many people due to too short night's sleep on working days and a delayed onset and prolonged duration of sleep on non-working days. Over 80% of people in western industrialized countries show this altered sleep behavior and that nightly sleep on non-working days is delayed by an average of 90 min (28).

Since circadian rhythms only have an approximate period length of 24 h, they require daily synchronization with the environment (29, 30). The rotation of the earth and thus the regular light-dark change is the most important environmental signal for the synchronization of circadian rhythms (31). For the interpretation of ambient brightness, photoreceptors continuously calculate the intensity, and spectral composition of the light entering our eyes (32). Thus, when light with increased short-wave radiation enters the eye, ipRGCs signal a bright phase of day to the SCN. In addition, the times of change from dark to light phase and vice versa (i.e., dawn and dusk) provide a crucial input for the SCN and the synchronization of circadian rhythms with the environment (31, 33, 34). Bright white light at night can shift the circadian phase backward by up to 3 h in the next 24-h cycle. In contrast, early morning exposure to light can shift the circadian phase forward by up to 2 h in the next 24-h cycle (35, 36).

Some studies document acute effects of bright light on the subjective feeling of alertness at night and during usual sleep periods (37–39), before falling asleep (40) and immediately after waking up in the morning (41, 42) but also during daytime (43). Although, most of these studies compare very low illuminances (5–50 lx) with high illuminances (1,000–5,000 lx) of fluorescent white light, alerting effects were estimated to occur already at around 100 lx during the night and at 500 lx during the evening. Thus, there is evidence, that subjective wakefulness due to bright light can basically occur at any time of the day. Other research with objective measures has so far only been able to prove a wakefulness inducing effect of bright light at night (44) and results of studies conducted during the day provided inconsistent results (43, 45, 46), possibly due to smaller differences in the light levels compared. Nevertheless, bright light during the day makes the circadian system less sensitive to nocturnal light (26, 47–49). Although the protocols of these studies differ from each other, illuminances that were compared during the day were significantly different from each other (i.e., at least 10-fold up to 400-fold different). A dark phase of several hours can increase sensitivity to light. Thus, early morning exposure to light immediately after awakening (i.e., after several hours of nightly sleep in darkness) can phase advance the circadian phase by 1–3 h (50, 51).

In many cases, the alerting effect of light, especially in the evening and at night, is also associated with an increase in attention and working memory performance (41, 52). Non-visual effects also include the mood enhancing effect of bright light (53–55), with required light doses being around 2500 lx-h. There is also evidence that the current mood reflects a person's level of alertness and the immediate effect of bright light on mood is mediated by the wakefulness inducing effect (56).

## Lighting Concepts That Address Non-Visual Effects of Light

Considering our experience with around 100 years of electric lighting, people have been exposed to this artificial creation 5,000 times shorter than to the light at night from fire. The first evidence for the use of handcrafted light sources comes from archaeological findings from around 500,000 years ago. Fire was used as a source of light at night, but life and work still depended on daylight. Daylight is perhaps the purest form of human centric lighting since our eyes have had several million times longer to optimize to daylight than to LEDs. It is therefore reasonable to assert, from the evolutionary standpoint, that human eyes, and behavior are not yet optimized to electric light.

New lighting technologies that try to mimic continuously changing CCT and illuminance of sunlight according to the time of day are often termed HCL (Human Centric Lighting). According to manufacturers of these lighting technologies, it is possible to provide people indoors with artificial light similar to daylight in such a way that they can benefit from the beneficial effects natural daylight would provide. These include increased alertness, concentration, and performance. A publication summarizing the benefits of HCL on humans shows that it has sound motivations (57). The authors conclude that “bright days and dark nights are a good starting point,” and suggest that apart from electric lighting, architecture should be driven by daylight design principles. A conclusion that we support. Since HCL is increasingly being promoted and used for work places or private homes due to their postulated effects, the Swiss State Secretariat for Economic Affairs (SECO), and the Federal Office of Public Health (FOPH) have commissioned the Centre for Chronobiology at the University of Basel to evaluate scientific literature on HCL (58). The central question was whether this light can influence physiological, cognitive, or subjective effects in humans, i.e., the effects perceived by humans themselves.

The Basel study (58) has shown that only a few studies have investigated whether HCL can influence the above mentioned effects. Therefore, the University of Basel has extended the assessment and additionally evaluated studies on physiological, cognitive, or subjective effects of artificial light that affects people during the day during office hours (from 7:00 a.m. to 5:00 p.m.) but does not continuously adapt to the properties of daylight. A total of 45 studies met the inclusion criteria. On the basis of these studies, it was possible to check for 33 different effect variables whether they depend on the light intensity and CCT of artificial light that affects people during the day.

The Basel study (58) shows that neither the light intensity nor CCT significantly influence physiological parameters such as pulse rate and brain waves during normal office hours. In the case of cognitive effects, however, it was shown that light intensity and CCT had an influence on the reaction time of people. In addition, the spectrum of light influences the accuracy with which people solve tasks. In the subjective effects, light intensity and light spectrum had an influence on the concentration, tiredness and drowsiness perceived by the persons themselves. Overall, however, the observed effect strengths of the light effect during office hours were rather small. Nevertheless, the authors of the study come to the conclusion that high light intensity and higher CCT during daytime hours are advantageous in artificially illuminated interiors, even if these advantages are only evident in cognitive and subjective effects but not in physiological parameters. During the night, the effects of higher CCT are more prominent even in field studies. While blue-enriched white light sources can adjust circadian rhythm to night-shiftwork, reduce sleepiness, and enhance cognitive performance of night-shift workers (59) this concept should be applied cautiously and only if workers must work very concentrated (e.g., in control rooms).

While there are numerous studies providing evidence of non-visual effects of light during the evening and at night, results might not be translatable to the day. In line with the Basel study (58), a literature review on daytime non-visual effects of light on alertness (60) concludes that the present literature provides inconclusive results on alerting effects of light during daytime, particularly for objective measures and correlates of alertness. The authors suggest that the alerting potential of exposure to more intense white light should still be investigated. Another systematic review assessed effects of light on alertness and mood in daytime workers (61). Although, they conclude that light with a high CCT may improve alertness during the day, they suggest that additional studies are still needed because all findings are based on low-quality evidence.

Impacts of two dynamic LED lighting concepts with illuminance and CCT gradually decreasing between 1:30 p.m. and 5 p.m. were investigated on its effects on sleep and well-being (62). In one setting illuminance changed from 700 to 500 lx and CCT from 6000 to 3500 K, in the other from 500 to 300 lx, and CCT from 5000 to 3000 K. The settings were compared to static light (500 lx, 5000 K and 300 lx, 4000 K). A significant increase in subjective alertness was observed at 1 p.m., indicating a potential solution to reduce the subjective sleepiness in the afternoon. On the other hand, a significant decrease in perceived sleep quality and sleep duration was reported after subjects were exposed to dynamic lighting. No significant differences were observed for mental stress, productivity, visual comfort, or perceived naturalness.

A different approach with custom-built desktop luminaires intended to support office occupants' entrainment while supporting their alertness during the day. The luminaires were designed to deliver three lighting interventions. First saturated blue light (455 nm, 50 lx) in the morning (6–12 a.m.), Then polychromatic white (6500 K, 200 lx) light at midday (12 a.m.−1:30 p.m.) provided a smooth transition from the first to the third intervention. The third intervention was saturated red light (634 nm, 50 lx) in the afternoon (1:30–5 p.m.). In their results the authors observed advances in sleep start and sleep end times and hence they suggest that the participants were better entrained to the local 24-h light dark cycle while at the same time reporting increased subjective alertness in the afternoon with red light (63).

The effect of dynamic light during shift work on the quality of sleep and melatonin secretion was examined with staff of an Intensive Care Unit (ICU) and compared with staff from a similar ICU with standard light (64). CCT controlled ceiling luminaires with light tubes (2700 and 6500 K) and indirect lighting with RGBW lights “to imitate the reflection of the sun” were used but no information about the spectral characteristics is reported. The light changed color and intensity. The nightlight between 10 p.m. and 5 a.m. was dim (68 lx), and short-wavelength depleted causing the light to appear “unnaturally red.” Between 5 and 6 a.m., the light gradually changed to a daylight scenario (525 lx). In the afternoon from 3 p.m. light levels decreased. Between 8 and 10 p.m. a change occurred “toward a mix of primarily red, green, and white.” Since no precise spectral measurements are available but only RGB percentages it is difficult to replicate the lighting conditions. Nevertheless, the intervention group reported to be more rested and assessed their condition on awakening as better than the control group. The study, however, found no significant differences in sleep efficiency and melatonin levels. Subjectively, nurses from the intervention group assessed their sleep as more effective than participants from the control group. In a different field study, bright fluorescent lighting (1500–2000 lx) when compared to standard lighting (300 lx) in hospitals decreased sleepiness of ICU nurses working a 10-h night shift (65).

In a field experiment, effects of dynamic lighting on office workers were tested (66). In the dynamic lighting condition, employees experienced a gradually changing lighting scenario (changing twice a day between 8 and 12 a.m. and 1:30 and 4 p.m. from 700 to 500 lx and 4700 to 3000 K). The static condition provided an illuminance of 500 lx and CCT of 3000 K While employees were more satisfied with the dynamic lighting there were no significant differences for need for recovery, vitality, alertness, headache and eyestrain, mental health, sleep quality, or subjective performance.

Under strictly controlled laboratory conditions, we examined whether dynamic light across the day influences cognitive performance, visual comfort, melatonin secretion, sleepiness, and sleep (67). Volunteers either woke up with static daylight LED (100 lx at the pillow and 4000 K, melEDI 69 lx) or with a dynamic daylight LED that changed CCT (2700–5000 K) and intensity (0–100 lx at the pillow, melEDI 0.4–76 lx) across the day (daylight here refers to the spectral characteristics of the Toshiba TRI-R LED). Participants underwent a 49-h laboratory protocol. They spent the first 5-h in the evening under standard lighting, followed by an 8-h nocturnal “baseline” sleep episode at habitual bedtimes. Thereafter, they spent a scheduled 16-h waking day under one of the lighting conditions. Following a 8-h nocturnal “treatment” sleep episode, the volunteers spent another 12 h either under static or dynamic light. Horizontal illuminance at desk height ranged, depending on the position, between 150 and 650 lux, which corresponds to standard office lighting. Under dynamic light, evening melatonin levels were less suppressed 1.5 h prior to usual bedtime, and participants felt less vigilant in the evening compared to static light. Sleep latency was significantly shorter compared to the static light condition while sleep structure, sleep quality, cognitive performance, and visual comfort did not significantly change. These results support the recommendation of using blue-depleted light and low illuminances in the late evening, which can be achieved by a dynamically changing LED solution. Since illuminance decreased to around 1 lx, this lighting concept can only be applied in domestic areas but if concentration at work in the late evening and at night is required, this lighting concept would be counterproductive.

To date, only a few field studies investigated the influence of dynamic lighting solutions during shift work. A field study surveying the state of subjective alertness and fatigue in 542 employees during three-shift work (8 h working shifts) in ongoing production operations compared dynamic light with a static lighting condition (68). In a first round, 256 respondents evaluated the static lighting concept by completing a structured questionnaire. In a second round, 287 respondents commented on the alternating lighting concept. Fourty one percent of the participants who experienced the alternating lighting concept took part in the survey on the static lighting concept. They worked in three shifts (morning, late, and night) for 8 h each. The alternating lighting concept featured a high horizontal illuminance on the workplace (850 lx) and a high CCT (5300 K) during daytime. The resulting vertical illuminance at eye height was 237 lx (melEDI 164 lx) and CRI was Ra 77. During nighttime, a reduced illuminance (580 lx) with low CCT (3400 K) and CRI of Ra 85 was deployed. This resulted in a vertical illuminance of 158 lx (melEDI 71 lx) at eye height. Illuminance and CCT changed gradually between 5 and 8 p.m. and between 5 and 9 a.m. This setting was compared to the static lighting condition (horizontal at the workplace 760 lx, vertical at the eye 210 lx (melEDI 128 lx), 4600 K, CRI Ra 82). All participants assessed specific lighting characteristics (such as CCT, brightness, color rendering, appeal) using a seven-point Likert scale. No significant differences were found between the participants' rating of the characteristics surveyed in terms of alternating and static lighting conditions. The transitions from day to night conditions and vice versa had no disturbing effect on the participants' rating of the lighting criteria surveyed (*p* > 0.05). Thus, it was concluded that shift workers accepted the alternating lighting system. In addition, there were no significant differences in alertness and fatigue between the early and late shifts for both lighting conditions. This survey indicated the potential usefulness of a dynamic lighting solution for shift-work without major impact on the worker's alertness and fatigue level. However, objective measures such as salivary melatonin levels, and reaction time measures to assess vigilant attention are clearly mandatory for future studies to test the usefulness of dynamic lighting solutions in shift work environments.

With the goal to provide adequate light for visual tasks while lessening disruption of the human circadian system, Moore-Ede et al. (69) derived a spectral sensitivity curve with a peak at 477 nm and a full-width half-maximum of 438 to 493 nm. While there are other products commercially available that notch the spectrum in the melanopsin region, they specifically call it “steadystate circadian potency spectral sensitivity” and suggest that it “permits the development of spectrally engineered LED light sources to minimize circadian disruption and address the health risks of light exposure at night in our 24/7 society, by alternating between daytime circadian stimulatory white light spectra and nocturnal circadian protective white light spectra.” They further suggest, that it could provide attractive and energy-efficient white electric light that minimizes circadian disruption if violet LED dies with peak wavelengths of 410 to 420 nm replace the typical 450 nm blue peak emissions of conventional LEDs. Since short-wavelength light is known to have alerting-, performance-, and mood enhancing properties (70, 71), they suggest that this light at night could be used to reduce human error, without the risk of circadian disruption and health disorders. The alerting effect of short-wavelength light could be retained because there is evidence by a single study that the alerting effects of 420 nm violet light are even greater than 440 or 470 nm blue light (72).

In an approach to mimic certain aspects of daylight (i.e., direct warm sunlight and diffuse cool skylight), Aalborg University proposed a combination of directional task lighting and diffuse ambient lighting, with respective intensities and CCTs to create naturally perceived luminous variations. Such lighting concepts mimicking the combination of light from the sun and sky date back to 1952 (73). Aalborg University conducted a pilot study with four participants that worked for 4 months in such static and dynamic lighting. Visual comfort, perceived atmosphere, and work engagement were evaluated with interviews and questionnaires. The tentative results indicate that dynamic lighting has a positive effect on visual comfort, perceived atmosphere, and work engagement compared to static lighting (74). Another study (75) from the same author investigated the quality of light in an office after adding ceiling-mounted spotlights to traditional diffuse ceiling panels with the intention to complement the directionality of the natural daylight inflow from windows. The visual light quality and perceived atmosphere of the office environment was tested with 30 volunteers through questionnaires, reaction cards and semi-structured interviews. The authors report: “The direct flow of light is recommended to be more than 15% of the total illuminance at the work-plane to provide the distinct visual appearance of modeling and a cozier atmosphere, which is preferable for socializing, and <45% to avoid glare and high contrast for visual tasks. Direct warm and diffuse cool lighting were perceived as the most natural but were not always preferred. There is a slight preference for cooler ambient lighting in clear sky situations and warmer ambient lighting in overcast situations. Strong individual preferences for combinations of color temperatures was identified….”

Effects on well-being and motivation by changing light distribution were investigated by Fleischer (76) in 2001. Lighting consisted of luminaires that slowly changed between direct and indirect light. The ratio between direct and indirect lighting was changed according to the time of the day or to weather conditions. It was shown that pleasure rises with higher illuminance and a large indirect component. This might be due to a “sky-like” impression of the bright ceiling. The preference for a large indirect component was also found by Houser et al. (77) who report a subtle overall preference, when the indirect contribution to horizontal illuminance was 60% or greater. With an increase of the direct component and an increase of illuminance arousal rises. The direct component results in a darker ceiling but brighter desk. Apparently this contradicts the findings of (23) and (25) that find higher sensitivity of ipRGCs for light coming from above. The results of Fleischer, however, may not be explained by NIF effects (by ipRGCs) but solely by visual effects.

## Existing Recommendations for Non-Visual and Circadian Aspects

From a non-visual and circadian perspective, compliance with the above-mentioned standards cannot guarantee that enough biological active light reaches the eye (78). Some studies (45, 79, 80) report that corneal illuminance levels of at least 1000 lx for several hours are necessary to achieve non-visual effects during the day. Many of these studies investigated the effects of light therapy in the morning while others also found effects with illuminances ranging from 1000 to 1700 lx during various normal office hours (when compared to 165–200 lx). Thus, lighting standards addressing visual aspects are currently not designed to account for non-visual light effects during the day.

Today, an evaluation of the non-visual effectiveness of radiation is based on a radiometric characterization of the radiation entering the eye, and the corneally measured spectral irradiance is weighted with the spectral sensitivity of all five photoreceptors and referenced to the spectrum D65 (standard illuminant) (81). The International Standard of the International Commission on Illumination (CIE) CIE S 026:2018 (82) “CIE System for Metrology of Optical Radiation for ipRGC-Influenced Responses to Light” defines spectral sensitivity functions, quantities, and metrics to describe the ability of optical radiation to stimulate each of the five photoreceptor types (S-cone, M-cone, L-cone, rhodopsin, and melanopsin). This standard also denotes a quantity named the “melanopic equivalent daylight illuminance” (melanopic EDI or melEDI), that is expressed in Lux. The melanopic EDI of a light condition expresses how much daylight results in the same melanopic irradiance as the test light condition. Nowadays lighting projects often include temporal variations of the light, both spectrally and in terms of intensity. Lighting projects that consider the possible effects of changing light on people, try to optimize well-being. However, as of today there are no specific regulations. Recommended practices sprout everywhere but experts in the field criticize them.

Seven examples for recommendations for circadian lighting are (in alphabetical order):

1. Chartered Institution of Building Services Engineers (CIBSE) and Building Research establishment (BRE) Research Insight Circadian lighting (83).
2. CIE S 026:2018 (82) “CIE System for Metrology of Optical Radiation for ipRGC-Influenced Responses to Light.”
3. DGUV 215-210 “Non-visual Effects of Light on Humans” (84).
4. DIN SPEC 67600:2013-04 (85) (technical report) “Biologically Effective Lighting - Planning Recommendations.”
5. Recommendations for Healthy Daytime, Evening, and Night-Time Indoor Light Exposure (9).
6. UL DG 24480 (86) “Design Guideline for Promoting Circadian Entrainment with Light for Day-Active People.”
7. The WELL standard “CIRCADIAN LIGHTING DESIGN” and the update WELL v2 pilot (87).

### CIBSE and BRE Research Insight Circadian lighting

Based on a literature review (83) and their results from a field-study they suggest these tentative recommendations:

1. “From mid-morning until early afternoon, use higher than normal levels of light with increased blue light. Current high color temperature light sources such as LEDs and some types of fluorescent light give high outputs of blue light. There is still scope to tailor their spectra further in the future to fit the peak response of the ipRGC sensors in the eye and maximize their circadian impact.
2. Toward the end of the day, dim the lighting (while retaining enough light to meet visual task recommendations) and lower its color temperature (“warmer,” redder light, similar to that in a domestic setting). There is also future scope to alter the spectrum of existing LEDs to give very low circadian stimulus in the evening or at night. Even warm white LEDs often have a small peak of blue light which can stimulate the ipRGCs.
3. Maximize reflected light from room surfaces by using light fittings with an upward light component, and “wall washing” to illuminate the walls directly. This will give more light to people facing the walls.
4. As light levels will be higher than normal for part of the day, use high quality fittings to minimize glare and avoid all flicker. Have a balanced visual environment, for example by avoiding very light-colored desks.
5. Vary the lighting gradually, to avoid disturbing the occupants. Controls need to be reliable.
6. People vary in their preferences for lighting; conventional good practice is to offer individual control but this can negate the circadian effects. There is no obvious way round this.
7. Explain to the occupants what the lighting system is doing and the purpose of varying the lighting.”

### CIE S 026:201861 “CIE System for Metrology of Optical Radiation for ipRGC-Influenced Responses to Light”

CIE recommends to spend adequate time outdoors during the day since it is associated with better health and well-being and also recommends to not restrict daylight within indoor settings. Although, no specific quantities are given, CIE recommends a high melEDI during the day to support alertness, the circadian rhythm, and good sleep during the night in a position statement on Non-Visual Effect of Light. During the evening and at night a low melEDI facilitates sleep initiation and consolidation (88).

### DGUV 215-210 “Non-visual Effects of Light on Humans”

This DGUV (German statutory accident insurance) information brochure (84) provides advice on hazards to safety and health at work, how they can be avoided and how opportunities for maintaining health can be exploited with modern lighting concepts. Since scientific knowledge about the non-visual effects of light on humans is not yet complete, as the brochure says, it is not yet possible to derive any generally valid quantitative statements regarding non-visual effects, for example numerical values for illuminance or CCT. This brochure gives the advice that daylight should be used first and foremost. For this reason, workplaces should preferably be located close to windows. The better the inner clock is synchronized by daylight, the less sensitive it is to disturbing factors, such as artificial light in the evening. Only if little daylight is available at workplaces, bright artificial lighting or lighting with high blue components should be used as a supplement during the day. Light sources with high CCTs are usually favorable for this purpose. This light can achieve similar non-visual lighting effects as daylight, but cannot replace it. In the evening, bright light and light with high blue components should be avoided. This should be done at least 2 h before the usual start of sleep. During this time, the light should primarily illuminate the work surface relevant to the visual task and not fall directly into the eye. Looking directly into the light source and at very brightly illuminated surfaces should be avoided. When working on a computer, tablet or smartphone, special blue light filter programs (e.g., flux, Night Shift or other manufacturer-specific blue light filter apps) should be used at least 2 h before the usual start of sleep. Furthermore, advice is given, that during the day, bright walls, and ceilings should enhance the non-visual effects through indirect light components. In the evening, the necessary brightness at the workplace should be limited. The lower indirect share of light on the ceiling and walls should reduce non-visual effects.

### DIN SPEC 67600:2013-04 “Biologically Effective Illumination - Design Guidelines”

The German DIN SPEC 67600:2013-04 “Biologically effective illumination - Design guidelines” recommends: Illuminance at the eye ≥ 250 lx at CCT = 8000 K or Illuminance at the eye ≥ 290 lx at CCT = 6500 K. The Commission for Occupational Health and Safety and Standardization (KAN) (represents occupational health and safety interests in the standardization process) criticizes (89):

“Contents of the already published DIN SPEC 67600:2013-04 (technical report) “Biologically Effective Lighting - Planning Recommendations” are partly based on insufficiently secured findings, therefore a misinterpretation during its application cannot be excluded … the planning recommendations of DIN SPEC 67600 (technical report) do not form a secure basis for the implementation of the Technical Regulation for Lighting ASR A3.4 in operation.”

CIBSE and BRE concluded in a literature review: “*The existing recommendations in DIN SPEC 67600 should be treated with caution.”*

### Recommendations for Healthy Daytime, Evening, and Night-Time Indoor Light Exposure

A recent publication (9) from experts in lighting, neurophysiological photometry and sleep and circadian research provides an expert consensus for healthy daytime and evening/night-time light environments. They come to the conclusion that “Throughout the daytime, the recommended minimum melEDI is 250 lx at the eye measured in the vertical plane at ~1.2 m height (i.e., vertical illuminance at eye level when seated). If available, daylight should be used in the first instance to meet these levels. If additional electrical lighting is required, the polychromatic white light should ideally have a spectrum that, like natural daylight, is enriched in shorter wavelengths close to the peak of the melanopic action spectrum. During the evening and at home, Brown et al. (9) recommend to reduce melEDI to around 10 lx at least 3 h before bedtime. During sleep the recommended maximum melEDI is 1 lx.

### UL DG 2448022 “Design Guideline for Promoting Circadian Entrainment With Light for Day-Active People”

UL DG 2448022 “Design Guideline for Promoting Circadian Entrainment with Light for Day-Active People” recommends: “The amount of light equivalent to that after 1 h of exposure, capable of suppressing the production of melatonin at night by 30 per cent … should be continuously available at the occupant's eyes for a minimum of 2 h during the daytime.” This would translate into a vertical illuminance at the eye of about 350 lx for warm light (CCT < 3000 K) and ~200 lx for cool light (CCT > 5000 K) sources. Here the question arises, how a suppression of melatonin at night relates to a measure of light during the day. UL DG 2448022 is commented by the IES (90) as follows:

“It is important to note that UL Design Guideline 24480 is not a consensus (ANSI) document. The IES maintains the position that any Recommended Practice related to light and health should be a consensus document developed through an accredited American National Standards Institute process. Without the full rigor of an ANSI approved Standard, non-consensus based information cannot be deemed to have been fully vetted and lacks the authority to provide public guidance regarding means or methods that affect public health. The IES urges the lighting industry to exercise caution when considering a non-consensus document for design, application, product qualification or regulatory purposes.”

### The Well Standard and Well v2 Pilot

The WELL standard recommends for melanopic light intensity at work areas: “Light models or light calculations demonstrate that at least one of the following requirements is met”:

1. At 75% or more of workstations, at least 200 equivalent melanopic lx (EML) is present, measured on the vertical plane facing forward, 1.2 m above finished floor (to simulate the view of the occupant). This light level may incorporate daylight, and is present for at least the hours between 9:00 a.m. and 1:00 p.m. for every day of the year.
2. For all workstations, electric lights provide maintained illuminance on the vertical plane facing forward (to simulate the view of the occupant) of 150 EML or greater.

The newer WELL v2 pilot recommends these levels for all spaces (at least 150 EML) and adds the corresponding EDI value (136 melEDI). In case that 218 melEDI or more are achieved, the space would achieve a better score in a system based on points. These light levels vertical plane at the eye should be achieved at least between the hours of 9 a.m. and 1 p.m. and may be lowered after 8 p.m. at night.

CIBSE and BRE concluded in a literature review (91): “*The existing recommendations in the WELL Building Standard should be treated with caution.”*

## Conclusion

The circadian timing system in humans is genetically timed in such way that we are active and awake during daytime and inactive and asleep during the night (i.e., diurnal species). Thus, during the biological night the hormone melatonin is actively secreted in a circadian fashion usually peaking 2–3 h after habitual bedtime. As melatonin is important for many physiological processes in the human body (e.g., antioxidant and regulating sleep-wake timing), its secretion should not be suppressed or altered in the evening and at night by light. Avoiding light at night during shift work, however, is rather difficult, especially if workers need to be fully concentrated. Thus, ideal lighting conditions during night shifts is always a trade-off between optimal light for visual tasks, safety, alertness, and well-being and optimal light for non-visual effects avoiding circadian phase shifts and melatonin suppression.

Exposure to brighter light and light with high proportions of short wavelengths in the blue spectral range during the day can improve subjective alertness, concentration, the reaction time, and accuracy with which individuals solve tasks. It reduces tiredness and drowsiness and helps to maintain circadian rhythms and improve sleep quality as compared to darker and blue depleted light. Physiological measures (e.g., EEG), however, are less likely to be affected by light during the day, most probably related to the fact that we are a diurnal species. The pathway of light for affecting circadian rhythms, sleep-wake behavior, alertness and well-being in individuals is predominantly expected to be through the eye and the stimulation of ipRGCs, which then send signals to the suprachiasmatic nucleus (SCN) and other areas in the brain implicated in the regulation of different neurobehavioral domains. Therefore, it can be assumed that melEDI is a suitable measure for predicting melatonin suppression and other non-visual effects in humans. A precise quantity of melEDI to trigger these effects however is difficult to determine and may depend on the output domain (e.g., alertness, melatonin, sleep, circadian phase shifts etc.,) one is interested in. Based on the expert consensus (9) mentioned above, we recommend to aim for 250 lx melEDI during usual daytime office hours (in the vertical plane at 1.2 m height) for everyone working inside buildings, even if this requires more energy.

Notably, luminous efficacy is calculated in Lumens per Watt because Lumens are based on visual brightness perception (V lambda curve). Non-visual aspects, however, should be considered too. Non-visual effects have a different spectral sensitivity curve compared to the visual perception of brightness and are therefore not considered in the common calculation of energy efficiency; therefore, we would recommend not only considering Lumens per Watt as a measure for luminous efficacy but also a measure that considers “non-visual luminous efficacy” during the day (e.g., melanopic EDI per Watt). When light is capable to reinforce normal circadian patterns of alertness during the day and sleep at night, studies have either used very high light levels (approximately 1000 lx) or strongly blue enriched light (CCT 17000 K). Notably, most field studies have not controlled for the position of individuals within a room and hence for their precise light exposure levels at the eye.

A lighting concept that reduces melatonin suppression during the night while still allowing for high concentration would be ideal. Since melatonin suppression is mainly linked to the melEDI (92, 93) and not necessarily to CCT, metameric light sources that reduce melatonin suppression could be an innovative solution (94). By optimizing the light spectrum metamerically, daytime alertness could also be improved (95). Additionally, the light distribution could be changed between night and day. Since direct light increases arousal (76) and iPRGCs are probably more sensitive to indirect light from the ceiling (25), these factors could be considered in the lighting design for day and night. Following existing guidelines to avoid glare and yet achieving a high amount of melEDIs at workplaces during the day could be achieved by three workarounds:

1. Optimizing the spectrum by using light sources with a relatively high melEDI (and a high CRI).
2. Optimizing vertical illuminances at the eye by optimizing the light distribution (also considering the reflection of surrounding surfaces. Optimized lighting design can provide higher light levels at the eye (vertical illuminances) for the same horizontal illuminance. Often, downwards oriented lighting from ceiling mounted luminaires intended for rooms with PC screen result in relatively low vertical illuminances. To achieve higher vertical illuminance and hence more light at the eyes, suspended or floor standing luminaires with indirect light distribution (that direct light onto the ceiling) and “wall washing” luminaires can be used. Additional white vertical elements can increase vertical illuminances.
3. Since the melanopsin-containing ganglion cells in the eye are distributed over a large area of the retina, it can be assumed that the non-visual effect of light is greatest when light comes from a large-area source. In nature, this light comes from the sky. If only a small area of the retina is illuminated, as is the case with the directional light of a spot, a weaker non-visual effect is assumed.

During the evening we share the opinion with Brown and colleagues (9) of reducing melEDI to around 10 lx, of course, only if no safety-relevant activities have to be carried out (i.e., at home before bedtime).

## Data Availability Statement

The original contributions presented in the study are included in the article/supplementary material, further inquiries can be directed to the corresponding author/s.

## Author Contributions

OS wrote the main manuscript text. CC provided critical review of and revisions to the manuscript. All authors contributed to the article and approved the submitted version.

## Conflict of Interest

The authors declare the following potential conflicts of interest with respect to the research, authorship, and/or publication of this article: OS is listed as an inventor on the following patents: US8646939B2—Display system having circadian effect on humans; DE102010047207B4—Projection system and method for projecting image content; US8994292B2—Adaptive lighting system; WO2006013041A1—Projection device, and filter thereof; WO2016092112A1—Method for the selective adjustment of a desired brightness and/or color of a specific spatial area, and data processing device thereof. OS is a member of the Daylight Academy. OS has had the following commercial interests in the last four years (2017–20) related to lighting: Investigator-initiated research grants from Derungs, Audi, VW, Porsche, Festo, ZDF, Toshiba, and SBB; Speaker fees for invited seminars from Merck, Fraunhofer, Firalux, and Selux. CC has had the following commercial interests in the last four years (2017–2020) related to lighting: honoraria, travel, accommodation and/or meals for invited keynote lectures, conference presentations or teaching from Toshiba Materials, Velux, Firalux, Lighting Europe, Electrosuisse, Novartis, Roche, Elite, Servier, and WIR Bank. CC is a member of the Daylight Academy.
